# Group-based intervention in a primary healthcare setting was more effective for weight loss than usual care

**DOI:** 10.4102/hsag.v24i0.1172

**Published:** 2019-09-16

**Authors:** Kathryn Manning, Marjanne Senekal, Janetta Harbron

**Affiliations:** 1 Department of Surgery, University of Cape Town, Cape Town, South Africa; 2 Department of Human Biology, University of Cape Town, Cape Town, South Africa

**Keywords:** Group-based intervention, Weight loss, Non-communicable diseases, Primary health care, South Africa

## Abstract

**Background:**

Literature and practice recommendations for lifestyle interventions to treat the increasing number of obese patients with non-communicable diseases (NCDs) or risk factors for NCDs attending resource-constrained public healthcare facilities in South Africa are scarce.

**Aim:**

To compare the impact of a facility-based therapeutic group (FBTG) intervention with usual care on weight in obese participants, with NCDs or risk factors for NCDs.

**Setting:**

Public healthcare facility providing primary healthcare services in Cape Town, South Africa.

**Methods:**

A quasi-experimental study design was used where participants chose to receive weight loss treatment with either the FBTG or usual care interventions. Both interventions involved a one-on-one medical and dietetic consultation, while FBTG participants had six additional group sessions. Follow-up assessments took place 6 months after baseline. Socio-demographic variables, blood pressure, smoking status, weight, height, waist circumference, dietary intake, physical activity and stage of change were measured.

**Results:**

Of the 193 obese adults enrolled, 96 selected the FBTG and 97 selected usual care. There were no significant differences at baseline between the two groups. Weight loss over 6 months was greater (*p* < 0.001) in FBTG (median [IQR] of −2.9 [−5.1; −0.3] kg) than usual care (−0.9 [−0.9; 0.6] kg) participants. At 6 months, more FBTG completers reached the weekly target of 150 min (*p* = 0.009), while both groups showed improvements in dietary intake. More FBTG (74%) than usual care (49%) participants were in the action stage of change by 6 months (*p* = 0.010).

**Conclusions:**

The group-based intervention was more effective than usual care in weight reduction as well as improvements in physical activity and stage of change.

## Introduction

Non-communicable diseases (NCDs) are becoming the greatest threat to human health and economic potential worldwide, especially in disadvantaged populations living in low- and middle-income countries (Kankeu et al. 2013; World Health Organization [WHO] 2014). The burden of NCDs such as diabetes mellitus (DM), cardiovascular diseases (CVD), stroke and cancer in South Africa is believed to be two to three times higher than that in developed countries (Mayosi et al. 2009). Furthermore, the rapid emergence of risk factors for NCDs, namely obesity, hypertension, hyperlipidaemia, unhealthy eating, physical inactivity, smoking and excessive alcohol intake, has been recognised as a significant public health challenge (Mayosi et al. 2012). There is uncertainty as to whether the country is prepared for the resource-demanding chronic care required to manage both the current and impending proportions of the population suffering from NCDs within a strained public healthcare (PHC) system (Levitt et al. 2011).

Within the South African context, NCDs and their associated risk factors have recently been identified as urgent intervention targets (Nojilana et al. 2016; South African Department of Health 2013), where undernutrition and infectious disease were the major priorities for many years. Despite this commitment, evidence-based guidelines for multi-level management of NCDs and obesity within PHC in low- and middle-income countries are still lacking. Furthermore, there is a paucity of research on lifestyle interventions that target obese adults with or without NCDs and risk factors in the culturally diverse South African population (Draper et al. 2010; Katz et al. 2009; Mash et al. 2014; Muchiri, Gericke & Rheeder 2015; Pengpid, Peltzer & Skaal 2014; Price, Shandu & Gill 2007; Puoane et al. 2007).

Current practice for dietary intervention for obesity or NCD management in PHC facilities in South Africa involves, at minimum, a once-off, one-on-one counselling session with a registered dietitian. Multiple dietetic follow-up sessions are mostly not feasible because of a high demand for dietary intervention, concurrent with insufficient human resources (Muchiri et al. 2015; Parker et al. 2012). It is thus highly likely that current management of obese patients and/or those with NCDs in PHC facilities is insufficient to make a clinically significant impact on patient outcomes.

Group-based programmes may offer a partial solution to this challenge because the approach is considered cost and time-saving for health professionals and facilitates intervention at a higher level of the socio-ecological model by including family and community members (Hoddinott et al. 2010). In developed countries, structured group-based, multi-focus lifestyle interventions for obesity and NCDs are widely accessible and have proved to be successful or at least equivalent to standard care in improving outcomes in a variety of settings and delivery modes (Kirk et al. 2012; Steinsbekk et al. 2012; Taggart et al. 2012; Venditti & Kramer 2012). In line with this approach, the Western Cape Chronic Disease Management (CDM) task team in South Africa developed guidelines and a model of care for a group-based NCD lifestyle intervention in 2009 with the aim to roll it out at both facility and community levels (Department of Health 2009; Draper, Draper & Bresick 2014). The model of care suggests that a PHC facility should provide a 6-week facility-based therapeutic group (FBTG) programme, followed by attendance of community support groups thereafter. The proposed FBTG programme was piloted in the Cape Metropole and some rural areas, but its impact was not tested in a controlled trial (Department of Health 2009).

The aim of this research was to determine the impact of a dietitian-led FBTG intervention on weight and body mass index (BMI) (primary outcomes) and waist circumference, dietary intake, physical activity, and stage of change (SOC) (secondary outcomes) in obese participants with NCDs or risk factors for NCDs.

## Methods and procedures

### Study design and study population

A quasi-experimental study design was used to achieve the stated aim. Following recruitment, volunteers were given the choice of the FBTG or usual care interventions. The study population was obese patients attending a PHC facility (False Bay Hospital) for their routine outpatient PHC medical appointments. False Bay Hospital is a district hospital that provides predominately PHC services in Cape Town. Participants were referred by medical doctors to the facility dietitian for a recruitment consultation. To be included, participants were required to be older than 18 years and have a BMI ≥ 30 kg/m^2^ with one or more intermediate risk factors for NCDs such as raised blood pressure (> 130/80 mmHg), raised HbA1c (> 7%), raised total cholesterol (> 4.5 mmol/L), and/or one or more existing NCDs, namely DM or CVD. A basic understanding of English was necessary as the interventions were conducted in English. Participants were excluded if they were unable to attend the FBTG sessions, were pregnant or lactating, or had any form of organ failure or severe psychiatric disorder, or were physically restricted.

During the initial recruitment consultation with the dietitian, both the FBTG and usual care options were presented to participants. Once participants decided on their preferred intervention, additional details of their chosen intervention and study procedures were provided; signed consent was obtained and baseline assessments were conducted. Participants who chose the FBTG intervention received information on dates and details of the six-session FBTG programme and were grouped according to their residential area in order to facilitate their enrolment in local community support groups thereafter.

The sample size estimation was based on the mean weight loss of 2.8 kg ± 4.0 kg (group-based participants) and 1.0 kg ± 2.9 kg (control) over 6 months as reported by Ash et al. (2006). To achieve 80% power at a 5% significance level, a sample of 60 per group was required. As attrition rates in weight loss interventions have been reported to be high (Moroshko, Brennan & O’Brien 2011), we aimed to recruit approximately 100 participants per group.

### Interventions

At baseline, all participants received the same routine medical consultation with a medical doctor and an initial 30-min consultation with the dietitian on diet and lifestyle for weight loss. All participants were scheduled for an individual follow-up consultation with the dietitian 6 months after baseline.

During the dietary consultation, both groups were encouraged to change their dietary patterns in line with the South African Food-Based Dietary Guidelines (Vorster, Badham & Venter 2013). The dietary guidelines were specifically tailored for weight loss and NCD prevention and management. All participants were advised to increase the intake of fruit and vegetables as allowed by the family budget (with the target to reach five portions a day); increase legumes and fish intake (specifically fish high in omega-3 fatty acids) to two or more times a week; and reduce energy-dense snacks, refined carbohydrates, added sugar and sugar-sweetened beverages (SSBs) to zero intake per day.

### The facility-based therapeutic group intervention

The FBTG intervention consisted of the baseline one-on-one consultation as mentioned, followed by the FBTG programme for 6 weeks (Table 1) and monthly community-based support groups thereafter until the 6 months follow-up assessments. The FBTG programme was based on the Western Cape NCD model of care (Department of Health 2009; Draper et al. 2014) and was designed to be participatory. Strategies of the Health Belief Model and socio-cognitive theory of behaviour change underpinned the intervention model and activities (Spahn et al. 2010). The chronic dispensing unit service (Du Plessis 2008) was provided to patients who met the criteria. The FBTG sessions were facilitated by a multi-disciplinary team consisting of the facility dietitian, pharmacist, physiotherapist and nurse over 6 weekly visits and were free of charge. The FBTG sessions ran on a weekday afternoon from 2 pm for a minimum of 60 min to a maximum of 90 min, which allowed for ± 30 min of education and ± 30 min for group discussion or practical tasks. On the day of the session, each participant received a short message service to remind them of their scheduled FBTG session.

**TABLE 1:** Curriculum for the six facility-based therapeutic group sessions.

Lifestyle component	FBTG sessions: topics, content and activities	Tools	Healthcare professional
**First session: Goal setting and nutrition education**			
Introduction	Introduction of clients and staff. Patients are given an overview of programme	-	Dietitian
Psychosocial counselling	Goal setting module Patients set individualised goals with the assistance of dietitian	CBT flip chart	Dietitian
Diet education	FBDG: Enjoy a variety of foods, Eat plenty of fruit & vegetables	FBDG flip chart	Dietitian
**Second session: NCD risk factors, problem-solving and nutrition education**			
Psychosocial counselling	Relaxation and breathing Understand, manage and control of NCDs and symptoms Health risk factors for NCDs: smoking, low physical activity and poor eating habits Assist patients to come to terms with current behaviour and to make better decisions to address the challenges	CBT flip chart	Dietitian
Diet education	Education on the following FBDG: Make starchy foods the basis of most meals† Use less salt Use fats sparingly† Issue meal plans according to energy requirements and assessments	FBDG flip chart Meal plans Example menu plan	Dietitian
**Third session: Internal and external locus of control, nutrition education and medication adherence**			
Psychosocial counselling	Understanding life, diseases and dealing with health challenges Managing life’s challenges and environments	CBT flip chart	Dietitian
Diet education	FBDG: Use food and drinks containing sugar sparingly and not between meals,† Drink lots of clean safe water, If you drink alcohol, drink it sensibly†	FBDG flip chart	Dietitian
Pharmacist education session	Medication adherence and details of the chronic dispensing unit. This service is used for chronic but stable patients who are encouraged to achieve stability in order to benefit from the shorter waiting times at the pharmacy. The session was also used as an opportunity for patients to ask questions about their prescriptions and discuss their personal tolerance of medications.	-	Pharmacist
**Fourth session: Problem-solving, and nutrition and physical activity education**			
Psychosocial counselling	Reframing your perspective on your health Problem-solving and changing habits	CBT flip chart	Dietitian
Exercise education	Education session: FBDG: Be active! Appropriate exercise for your NCD and injury prevention with a focus on breathing, pulse rate, fitness and the risks and benefits of exercising with certain NCDs.	FBDG flip chart	Physiotherapist
Diet education	FBDG: Eat dry beans, peas, lentils and soya regularly Chicken, fish, meat, milk or eggs can be eaten daily	FBDG flip chart	Dietitian
**Fifth session: Behaviour change, self-monitoring and physical activity session**			
Maintenance counselling	Making permanent lifestyle changes How to make changes permanent and the importance of self-monitoring	CBT flip chart	Dietitian
Diet education	Summary of FBDG and exercise goals	FBDG flip chart	Dietitian
Exercise education	FBDG: Be active! †Low-intensity exercises. This included a practical session, patients were expected to engage in low-intensity physical activity according to their ability	FBDG flip chart	Physiotherapist
**Sixth session: Maintaining changes and support**			
Maintenance counselling	Education session: The final FBTG session focused on the importance of patient driven community support groups for the management of NCDs and maintenance of healthy behaviours and weight loss. The session was either delivered by a registered nurse who had previously worked with diabetic support groups or by a support group coordinator from the local non-governmental organisation (NGO) for the False Bay area.	-	Registered nurse or support group coordinator

CBT, cognitive behaviour theory; FBDG, food-based dietary guidelines; FBTG, facility-based therapeutic group; NCDs, non-communicable diseases; NGO, non-governmental organisation.

† , Food-based dietary guidelines was adapted for obesity and NCDs.

The dietitian coordinated the multi-component FBTG programme and developed the curriculum for the six group sessions that focused on goal setting, healthy eating, physical activity, behaviour change and adherence, and advice on weight and behaviour maintenance (Table 1). Dietary education was provided in most sessions by the dietitian using the adapted South African Food-Based Dietary Guidelines (Vorster et al. 2013) and the plate model (United State Department of Agriculture 2011). Specific emphasis was placed on portion control, as well as reduction in unhealthy fats, sugar and convenience foods, and reinforcing the dietary recommendations provided in the initial individual consultation. The FBTG participants also received a food list with healthy and unhealthy food choices, a flexible meal plan that allowed participants to build their meals using portion-specific foods, and an example of a 1-week menu plan.

A flip chart for cognitive behaviour therapy, developed by psychologists, holistic practitioners and life coaches, was used during all FBTG sessions to address barriers for behaviour change (Department of Health 2009). The flip chart addressed issues such as poor goal setting, denial, victim and addictive behaviour and was used to encourage participants to discuss individual, social and environmental issues that could reduce their ability to change.

### Usual care

Usual care was defined as the current routine treatment provided to a patient with NCDs and/or risk factors for NCDs at False Bay Hospital. This includes initial one-on-one consultations with the medical doctor and dietitian. Follow-up appointments are usually scheduled with the medical doctor at least every 6 months, while dietetic follow-up consultations were offered on a monthly basis but were dependent on both the patient and dietitian’s availability.

## Measures

The dietitian took anthropometric measures, completed the interviewer administered questionnaire and accessed the facility database and participants’ folders to complete certain sections of the questionnaire.

### Baseline assessments

Socio-demographic characteristics included employment status, the number of years of formal education (successful years of schooling, diplomas and certificates), race (mixed ancestry, black African people, white and Asian people), gender (male and female) and date of birth. Family income was obtained and classified using the Western Cape provincial categories (refer to footnote in Table 3).

Smoking status was obtained from all participants at baseline. Fasting blood samples and blood pressure measurements were collected by the nursing staff at baseline to provide information needed for the inclusion and exclusion criteria, and to characterise the sample at baseline. The National Health Laboratory Service (NHLS) analysed the blood samples for glycated haemoglobin (HbA1c) and total cholesterol. An automated sphygmomanometer was used to measure BP. Medical doctors used the International Classification of Disease (ICD-10) codes to indicate diagnosis of hypercholesterolaemia, hypertension, DM and CVD (specifically ischaemic heart disease, heart failure and peripheral vascular disease) for each participant.

### Attendance

Attendance records for all participants were maintained rigorously throughout the 6-month intervention period. Reasons for non-attendance of dietetic appointments or FBTG sessions were obtained by telephone from participants who were contactable. Participants were considered lost to follow-up (LTFU) if they did not attend their final 6-month appointment. Completers were defined as participants with data for both baseline and 6-month collection points.

### Anthropometry

Weight and waist circumferences were measured at baseline, at each FBTG session and at 6 months using a calibrated electronic scale and a non-stretchable measuring tape, respectively. Height was measured at baseline using a stadiometer. All measurements were recorded to the nearest 0.1 kg and 0.1 cm as applicable. Body mass index was calculated as weight in kilograms (kg) divided by the height squared (in meters) and categorised according to the WHO classification (WHO 2006).

### Physical activity

Data on physical activity were collected at baseline and 6 months. Participants were asked whether or not they participated in formal physical activity, which was defined as activity that was intentional and of a higher intensity with the specific aim to promote physical fitness (Joubert et al. 2007). The number and duration of physical activity sessions per week or month were recorded and used to calculate the minutes of physical activity per week. Participants were categorised as inactive (no engagement in physical activity), insufficiently active (physical activity > 0 min, but < 150 min per week) and sufficiently active (≥ 150 min per week) (Joubert et al. 2007).

### Dietary intake

Dietary intake assessment was conducted at baseline and 6 months. The aim was to describe the proportion of patients who (1) achieved the recommended intake for nine indicator food groups and/or (2) experienced a positive change in intake of the indicator food groups over the 6-month intervention period (Table 2). For these purposes, a semi-quantified food frequency questionnaire (FFQ) consisting of 54 food items was developed (Manning, Senekal & Harbron 2016). The frequency of intake of a standard portion of each food item on the FFQ was recorded as the number of times the food was consumed per day, week or month. The number of standard portions consumed per day for each food item was calculated and then used to calculate the daily counts of standard portions consumed from each of the indicator food groups (Table 2). To measure the change in dietary intake over 6 months, the change in the proportion of participants who met (1) the recommended intakes based on food-based dietary guidelines (Vorster et al. 2013) that were tailored for NCDs and risk factors for NCDs or (2) an alternative practical cut-point that was more aligned with the usual intake of patients at the PHC facility was calculated. The practical cut-point was formulated to investigate the improvement in the intake of food groups for which the intake of the majority or all participants did not meet the recommended cut-points before and after the intervention. The practical cut-points were derived from the practical experience of the dietitian who had been providing dietary counselling to patients at the hospital for a period of 4 years at the time of study initiation.

**TABLE 2:** Indicator food groups derived from the food frequency questionnaire and the recommended and practical cut-points used in analyses.

Indicator food group	Items included (standard portion size)	Recommended intake†	Practical cut-point‡
Fruit and vegetables	Fresh fruit (1 tennis ball size); starchy vegetables (1/2 cup); cooked vegetables (1/2 cup), salad (1 cup)	≥ 5 portions a day (400 g per day)	≥ 3 portions a day
Legumes	Lentils, kidney/butter/broad beans, chick peas (1/2 cup)	≥ 2 portions per week	-
Fish (fresh or tinned)	Any white fish, tuna, pilchards, sardines (90 g)	≥ 2 portions per week	-
Energy-dense snacks	Baked goods (1 piece of cake, biscuits); crisps (1 packet); sweets (1 unit); chocolates (~50 g bar)	0 portions per day	≤ 3 portions per week
High fat foods	Fried chips (1 medium potato), fried chicken (90 g), fried fish (90 g), baked goods (1 portion), processed meat (2 slices), take-away foods (1 portion)	0 portions per day	≤ 3 portions per week
Refined CHO foods	White bread (1 slice); rice (1/2 cup) maize porridge (1/2 cup); jam (1 tsp); syrup (1 tsp)	0 portions per day	≤ 2 portions per day
Added sugar	White or brown cane sugar added to food or drink (1 tsp)	0 portions per day	-
Sugar-sweetened beverages	Fruit juice (125 mL); sugar-containing carbonated drinks (250 mL); cordials (250 mL)	0 portions per day	-

CHO, carbohydrate; tsp, teaspoon; mL, millilitre; g, grams.

† , Recommended intake cut-point based on meeting food-based dietary guidelines (Vorster et al. 2013) adapted for weight loss, NCDs and risk factors for NCDs.

‡ , Practical cut-points were derived from the practical experience of the dietitian who had been providing dietary counselling to patients at the PHC facility for a period of 4 years at the time of the study.

### Stage of change

To determine the participant’s readiness to increase their intake of healthy foods, an adapted version of the 12-item readiness for change questionnaire (RCQ) was completed at baseline and 6 months (Manning et al. 2016). The adapted RCQ categorises participants in either pre-contemplation, contemplation or action SOC and consists of nine statements, three per SOC category. The response options and scoring are as follows:−2, ‘strongly disagree’; −1, ‘disagree’; 0, ‘unsure’; +1, ‘agree’; or +2, ‘strongly agree’. These scores were summed to calculate a total score for each stage that could range from −6 to +6. The highest of the three scores indicates the SOC the participant is in. When the scores for two categories were equal, the participant was classified as being in the higher SOC of the two stages.

## Statistical methods

Data were cleaned and analysed using Stata version 14.2 (Stata Corporation, College Station, TX). Depending on the data distribution, numerical data were expressed as means with standard deviation or medians with interquartile ranges. Frequencies and proportions were used to describe categorical variables. Independent samples *t*-test (normal distributed numerical data) or Wilcoxon rank-sum test (non-normal distributed numerical data) was used to: (1) compare treatment groups (full baseline sample and completers) at baseline; (2) compare treatment groups (completers) at 6 months; (3) compare baseline variables between completers with participants LTFU and (4) compare the within-group change in numerical variables over the intervention period between the treatment groups. Wilcoxon signed-ranked tests were used to assess the change in numerical data over the intervention period within each treatment group. Pearson’s chi-squared test or Fisher’s exact test (if one or more cells had expected frequencies < 5) was used to compare categorical variables between treatment groups at baseline and 6 months. Exact test for symmetry for *k* × *k* contingency table and McNemar’s chi-square or exact test (for discordant pairs < 20) were used to assess the within-group change (from baseline to 6 months) for dichotomous paired data.

To determine the impact of the intervention on change in primary outcome variables (weight and BMI), per protocol analyses (data of completers only) and multiple imputation intention-to-treat (ITT) analyses were conducted to impute missing weight at 6-month follow-up in patient LTFU. Five imputations were performed using the multiple imputation by chained equations (MICE) with treatment group, age, race, gender, education, employment and baseline weight included in the imputation model. All results with a *p* < 0.05 were described as statistically significant.

### Ethical considerations

Permission to conduct the research was obtained from the Department of Health (DOH) and the medical superintendent of False Bay Hospital. Ethical approval was obtained from the University of Cape Town (UCT) Faculty of Health Science Human Research Ethics committee (Ref: 18/2010). Patients were only included in the study if they provided informed, written consent.

## Results

**FIGURE 1:**
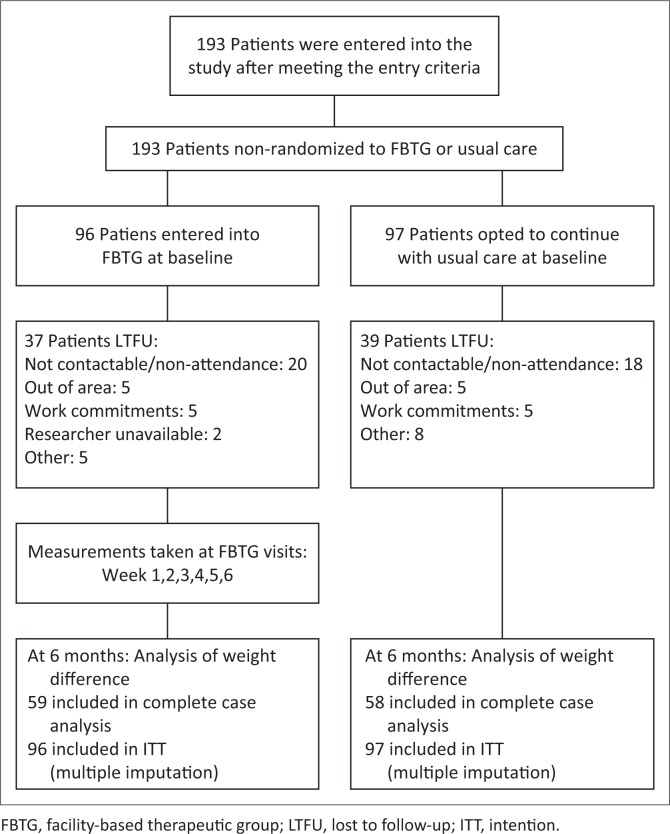
Flow of patients through study from baseline to 6 months.

Figure 1 illustrates the flow of participants through the study. The proportion of participants LTFU was 38.5% (*n* = 39) in the FBTG and 40.2% (*n* = 37) in the usual care group. Participants who were LTFU were significantly more educated (median [IQR]: 11.0 [10.0–12.0] years) than completers (10.0 [8.0–12.0] years) (Wilcoxon rank-sum test, *p* = 0.015). There were no other significant differences between completers and those LTFU (results not reported). Reasons for not attending the final 6-month follow-up appointment are summarised in Figure 1.

There were no significant differences in baseline characteristics between FBTG and usual care participants except for smoking status (Table 3). Over half of the sample in both groups had never smoked. The proportion of current smokers was significantly higher among usual care participants compared to FBTG participants.

**TABLE 3:** Socio-demographic variables, non-communicable diseases, intermediate risk factors for non-communicable diseases and smoking status by intervention group.

Variables	FBTG intervention (*n*† = 96)		Usual care (*n*† = 97)		Difference between treatment groups
	*n*	%	*n*	%	*p*
**Gender**					
Female	76	79.2	74	76.3	0.631‡
Male	20	20.8	23	23.7	-
**Race**					
Mixed ancestry	52	54.2	43	44.3	0.072‡
Black African people	17	17.7	31	31.0	-
White/Asian people	27	28.1	23	23.7	-
Currently employed (% yes)	48	50.0	57	58.8	0.222‡
**Family income per month**¶					
H0: (R0.00/government pension)	17	17.7	20	20.6	0.617§
H1: (< R4166.66 / < £359.85)	60	62.5	58	59.8	-
H2: (R4166.67 – R8333.33/£359.85 –£719.70)	14	14.6	10	10.3	-
H3: (> R8333.34/> £719.70)	4	4.2	5	5.2	-
Private (medical aid)	1	1.0	4	4.1	-
**NCDs and intermediate risk factor indicators**					
Diabetes (% yes)	36	37.5	45	46.4	0.211‡
Cardiovascular disease (% yes)	18	18.8	17	17.5	0.825‡
High blood pressure (% yes)	78	81.3	83	85.6	0.420‡
High cholesterol† (% yes)	57	65.5	64	73.6	0.249‡
**Smoking status**					
Never smoked	58	60.4	55	56.7	0.028‡
Current smoker	9	9.4	22	22.7	-
Previous smoker	29	30.2	20	20.6	-

FBTG, facility-based therapeutic groups; NCDs: non-communicable diseases.

† , Note: *n* varies because of missing values.

‡ , Chi-squared test used for comparison of categorical variables.

§ , Fisher’s exact test used for comparison of categorical variables with cell counts < 5.

¶ , H0, H1, H2 and H3 were the government classification for monthly income at time of assessment. H0 (ZAR0.00 or government pension), H1 (ZAR0.01-4166.66), H2 (ZAR4166.67-R8333.33), H3 (> ZAR8333.34) and P (private medical aid).

FBTG completers experienced a significant within-group reduction in weight and BMI over 6 months, while a non-significant reduction was evident for usual care completers (Table 4). The results of the impact analyses for the primary outcome variables in the complete case and imputed analyses confirmed that FBTG participants achieved significantly greater reductions in weight and BMI over 6 months compared to usual care participants (Table 4). The median (IQR) percentage weight loss over 6 months for FBTG and usual care completers was 2.4% (−0.6; −4.9) and 0.3% (−1.2; 2.9), respectively, *p* = 0.061. The median number of group sessions attended by FBTG completers was four, ranging between a minimum of one to a maximum of six sessions. The planned monthly community-based support group sessions were not rolled out because of unanticipated logistical constraints.

**TABLE 4:** Anthropometric measurements at baseline and follow-up, as well as comparisons between and within intervention group.

Variables	FBTG intervention			Usual care			Difference between groups	Change within groups	
								FBTG	Usual care
	*n*	Median	IQR	*n*	Median	IQR	*p*‡	*p*§	*p*§
**Height (m)**									
Baseline, full sample	96	1.61	1.57; 1.68	97	1.60	1.54; 1.67	0.292	-	-
**Weight (kg)**									
Baseline, full sample	96	100.8	88.0; 112.4	97	99.4	86.9; 115.8	0.863	-	-
6 months, imputed full sample	96	97.8	84.6; 109.6	97	98.0	86.4; 113.4	0.378	-	-
Baseline, completers	59	99.3	87.5; 108.8	58	102.0	86.3; 117.4	0.540	-	-
6 months, completers	59	96.5	84.6; 107.5	58	99.1	86.3; 115.3	0.304	-	-
6 months minus baseline¶	-	-	-	-	-	-	-	-	-
Imputed full sample analysis	96	−2.9	−5.1; −0.3	97	−0.9	−2.9; 0.6	< 0.001	< 0.001	< 0.001
Complete case analysis	59	−2.5	−5.2; 0.6	58	−0.4	−2.6; 1.2	0.041	< 0.001	0.070
**BMI (kg/m2)**									
Baseline, full sample	96	37.7	34.1; 43.1	97	38.6	33.7; 43.4	0.591	-	-
6 months, imputed full sample	96	36.0	33.1; 41.9	97	37.6	33.7; 42.6	0.210	-	-
Baseline, completers	59	37.4	33.2; 42.0	58	36.6	33.7; 43.2	0.556	-	-
6 months, completers	59	35.9	32.7; 40.0	58	37.2	33.3; 42.6	0.278	-	-
6 months minus baseline¶	-	-	-	-	-	-	-	-	-
Imputed full sample analysis	96	−1.1	−2.0; −0.1	97	−0.3	−1.2; 0.2	< 0.001	< 0.001	< 0.001
Complete case analysis	59	1.0	−0.2; 2.2	58	0.1	−0.5; 1.1	0.043	< 0.001	0.070
**Waist circumference (cm)**									
Baseline, completers	59	114.0	107; 124	58	117.0	110; 125	0.230	-	-
6 months, completers†	58	110.5	104.0; 120.0	57	117.0	109; 123	0.017	-	-
6 months minus baseline¶	58	−4.0	−6.0; 0.0	57	0.0	−3.0; 1.0	< 0.001	< 0.001	0.423

FBTG, facility-based therapeutic groups; IQR, interquartile range; BMI, body mass index; WC, waist circumference.

† , *n* varies because of missing values.

‡ , Wilcoxon rank-sum test used to compare non-normally distributed data between FBTG and usual care.

§ , Wilcoxon signed-rank test used for non-normally distributed paired data to compare change from baseline to follow-up within each treatment group.

¶ , 6 months minus baseline reflects the change in weight, BMI and WC over 6 months.

FBTG completers experienced a significant within-group reduction in waist circumference (WC) over 6 months, which was significantly greater than the non-significant reduction experienced by usual care completers (Table 4).

Most participants in both treatment groups were not participating in formal physical activity and were classified as inactive at baseline (Table 5). At 6 months, a significantly larger proportion of FBTG completers partook in formal physical activity and met the recommended target of >150 min of formal physical activity per week compared to usual care completers.

**TABLE 5:** Participation in formal physical activity and level of physical activity at baseline, 6 months and change over 6 months (completers).

Variables	FBTG intervention *n* = 59†		Usual care *n* = 58†		Difference between groups	Change within groups	
						FBTG	Usual care
	*n*	%	*n*	%	*p*‡	*p*	*p*
**Participating in physical activity**							
Baseline completers (% yes)	10	17.2	8	13.8	0.608	-	-
6 months (% yes)	22	40.0	9	16.1	0.005	0.002§	0.706§
**Physical activity level at baseline**							
Inactive	48	82.8	50	86.2	0.515	-	-
Insufficiently active	6	10.3	7	12.1		-	-
> 150 min per week	4	6.9	1	1.7		-	-
**Physical activity level at 6 months**							
Inactive	33	60.0	47	83.9	0.007	-	-
Insufficiently active	13	23.6	8	14.3		-	-
> 150 min per week	9	15.3	1	1.72		0.007¶	1.000¶

*Source*: Physical activity categories devised from Joubert, J., Norman, R., Lambert, E.V, Groenewald, P., Schneider, M., Bull, F. et al., 2007, ‘Estimating the burden of disease attributable to physical inactivity in South Africa in 2000’, *South African Medical Journal* 97(8), 725–731, viewed 21 July 2009, from http://www.ajol.info/index.php/samj/article/view/13903.

† , *n* varies because of missing values.

‡ , Chi-square or Fisher’s exact test used for comparison of physical activity categorical variables.

§ , *p*-value for within-group change in FBTG completers (*n* = 51) and usual care (*n* = 54) with paired data over 6 months using McNemar’s test (chi-square or exact) for 2 × 2 comparisons.

¶ , Exact test for symmetry for *k* × *k* contingency table.

A small number of completers consumed the recommended ≥ 5 portions of fruit and vegetables per day (FBTG: *n* = 6 [baseline] and *n* = 5 [6 months]; usual care: *n* = 1 [baseline] and *n* = 4 [6 months]) (Table 6). When applying the practical cut-point of ≥ 3 portions of fruit and vegetables per day, a significantly larger proportion of usual care completers (50.9%) consumed this target at 6 months compared to baseline (29.8%) (Table 6).

**TABLE 6:** Dietary intake from indicator food groups (portions per day) at baseline, 6 months and change over 6 months.

Variables	FBTG (*n* = 59†)		Usual care (*n* = 58†)		Difference between groups	Change within groups	
						FBTG	Usual care
	*n*	%	*n*	%	*p*‡	*p*§	*p*§
**Fruit and vegetables, consumed ≥ 3 per day**							
Baseline	26	46.4	17	29.8	0.069	-	-
6 months	28	52.8	28	50.9	0.842	0.394	0.022
**Legumes, consumed ≥ 2 per week**							
Baseline	16	28.6	12	21.1	0.355	-	-
6 months	23	43.4	16	29.1	0.122	0.096	0.359
**Fish, consumed ≥ 2 per week**							
Baseline	12	21.4	8	14.0	0.303	-	-
6 months	17	32.1	8	14.6	0.031	0.157	0.763
**Energy-dense snacks, consumed ≤ 3 portions per week**							
Baseline	16	28.6	26	45.4	0.061	-	-
6 months	26	49.1	36	65.5	0.085	0.016	0.064
**High fat foods, consumed ≤ 3 portions per week**							
Baseline	25	44.6	23	40.4	0.644	-	-
6 months	38	71.7	31	56.4	0.097	0.002	0.096
**Refined CHO foods, consumed ≤ 2 portions per day**							
Baseline	7	12.5	5	8.8	0.557	-	-
6 months	19	35.9	10	18.2	0.038	0.002	0.125
**Added sugar, consumed 0 portions per day**							
Baseline	23	41.1	14	24.6	0.062	-	-
6 months	34	64.2	21	38.2	0.007	0.007	0.016
**SSBs, consumed 0 portions per day**							
Baseline	32	57.1	32	55.2	0.832	-	-
6 months	35	66.0	33	60.0	0.516	0.167	0.754

FBTG, facility-based therapeutic groups; CHO, carbohydrates; SSB, sugar-sweetened beverages.

† , Note: *n* varies because of missing values either at baseline or 6 months.

‡ , Chi-square test used for comparison of dietary categorical variables between groups.

§ , McNemar’s test used for comparing the within-group change in FBTG completers (*n* = 51) and usual care (*n* = 54) with paired data over 6 months.

FBTG, facility-based therapeutic group; LTFU, lost to follow-up; ITT, intention.

Most completers in both groups did not consume the recommended intake of zero portions per day for refined carbohydrate (FBTG: *n* = 0 [baseline] and *n* = 2 [6 months]; usual care: *n* = 0 [baseline and 6 months]), energy-dense snacks (FBTG: *n* = 1 [baseline] and *n* = 6 [6 months]; usual care: *n* = 2 [baseline] and *n* = 6 [6 months]) and high fat foods (FBTG: *n* = 1 [baseline] and *n* = 5 [6 months]); usual care: *n* = 1 [baseline] and *n* = 7 [6 months]). A significantly larger proportion of FBTG completers consumed ≤ 2 portions per day of refined carbohydrate foods at 6 months compared to baseline (Table 6). Furthermore, a significantly larger proportion of completers in both groups consumed ≤ 3 portions energy-dense snacks and high fat foods per week, as well as zero added sugar per day at 6 months compared to baseline. At 6 months, a significantly larger proportion of FBTG completers consumed ≥ 2 portions fish per week, ≤ 2 portion refined carbohydrates and zero added sugar per day compared to usual care completers (Table 6).

**FIGURE 2:**
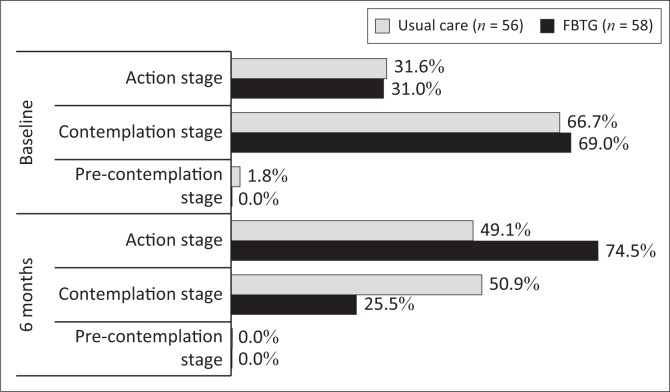
Stage of change between treatment groups (completers) at baseline and 6 months.

By 6 months, significantly more FBTG completers were in the action stage and fewer were in the contemplation stage compared to usual care completers (Figure 2). The proportion of participants in the action stage increased by 43.5% in the FTGB group and only 16.5% in the usual care group.

## Discussion

The results of this study show that participants who received a dietitian-led FBTG intervention at a PHC facility in Cape Town that provides PHC services experienced greater reductions in weight (2.0 kg) and BMI (0.8 kg/m^2^) (primary outcomes) and improvements in several secondary outcomes compared to participants who received usual care. The weight reduction of 2.9 kg that amounted to 2.6% of body weight in the FBTG completers over the 6-month intervention period is within the range of −1.3 to −8.2 kg reported in a systematic review on group-based interventions for NCDs of approximately 6-month duration (Gallagher et al. 2013). However, it is below the recommended ≥ 5% that is associated with clinically meaningful reductions in NCDs or intermediate risk factors for NCDs (Wing et al. 2011). When compared to literature, the intervention dose of six sessions over 6 months may have been insufficient and could explain the more limited weight loss results in our study. It is evident from the review by Anderson, Luan and Hoie (2004) that the number of sessions provided in various dietary interventions ranges from 10 to 22 over 6 months. The literature and our results thus indicate that an intervention intensity of more than six sessions with the inclusion of ongoing support and reinforcement post-intervention for a minimum of 12 months is necessary to achieve clinically significant weight and behavioural change (Clark et al. 2010; Kirk et al. 2012; Venditti & Kramer 2012). The fact that weight loss experienced by the usual care group was not significant reflects the possibility that the current usual care delivered at PHC facilities may not be sufficient for treatment or management of obesity.

Waist circumference reduction was consistent with weight loss in the FBTG completers, while no significant changes occurred in the usual care group. The 3.7 cm reduction in WC experienced by the FBTG completers is comparable with the WC reduction experienced by participants in other group-based interventions (Gallagher et al. 2013). A reduction in central adiposity may result in lower risk of metabolic problems and NCDs such as DM and CVD and is thus strongly recommended for patients who have one or more NCDs or risk factors for NCDs (WHO 2008).

The FBTG intervention was effective in increasing the formal physical activity levels of FBTG completers over the 6-month intervention period. However, only a minority of completers were able to meet the recommended 150 min of moderate-intensity activity per week for general health benefits and/or to achieve 1% – 3% weight loss for adults aged 18–64 years (WHO 2010). It is possible that the improvement in physical activity levels observed in FBTG participants was a result of the two physical activity sessions conducted by a physiotherapist as part of the FBTG intervention, as well as the motivation and behavioural strategies provided throughout the programme, which are not typically part of usual care. In a meta-analysis of physical activity interventions for obese individuals, Gourlan, Trouilloud and Sarrazin (2011) reported that a dosage of 37 sessions was strongly associated with increased, clinically meaningful levels of physical activity. Although this level of input is far beyond what could be achieved in a programme rolled out in a PHC facility, the importance of inclusion of some level of physical activity education in PHC intervention programmes is unquestionable, and an increase in intervention intensity (number and components focusing on physical activity) may assist participants in meeting physical activity targets. Low levels of physical activity in South African urban communities are promoted by factors such as lack of safe, green recreational space and reduced occupational and transport-related physical activity (Peltzer & Phaswana-Mafuya 2012). These factors should be addressed with population-level interventions (WHO 2010).

Overall, both groups seemed to have benefitted from exposure to the dietary advice received in terms of improvements in the intake of ‘obesogenic’ indicator food groups such as the energy-dense snacks, high fat foods and added sugar. In addition, FBTG completers were successful in decreasing their intake of refined carbohydrates. However, the intake of SSB did not improve in either group. The improvements in refined carbohydrate and added sugar intake per day were significantly greater in the FBTG than usual care completers. It is possible that reinforcement of dietary messages and behavioural change strategies included in the FBTG sessions contributed to this result. It remains a concern that the consumption of added sugar and SSBs was still very prevalent at the conclusion of the study. Approximately one-third of FBTG completers and two-thirds of usual care completers consumed added sugar on a daily basis and approximately one-third of completers in both groups consume SSBs on a daily basis at 6 months. Of the participants in the South African National Health and Nutrition Examination Survey (SANHANES) (Shisana et al. 2014), 58% had a moderate to high sugar intake score, as well as the reported high intake of SSB in the South African population (Vorster et al. 2014). The consumption of one to two SSBs per day has been found to be associated with increased risk of DM, metabolic syndrome, heart disease (Temple & Steyn 2013), risk factors for NCDs (Vorster et al. 2014) and weight gain (Malik et al. 2013). A more intense focus on behaviour change strategies that may facilitate change in sugar and SSB consumption is clearly indicated in interventions targeting patients with NCDs or NCD risks attending secondary healthcare facilities in the study area.

Changes in the intake from the healthy indicator food groups were also observed in both groups. Significantly more FBTG compared to usual care completers consumed the recommended number of portions of fish at 6 months. However, despite this improvement, more than half of FBTG completers still did not achieve the recommended > 2 portions of fish per week. Usual care completers, on the other hand, experienced a significant improvement in fruit and vegetable intake that was not evident in the FBTG completers, indicating that the information provided in the individual consultation with the dietitian may have an impact in this respect. Low levels of fruit and vegetable intake have been described in South Africans (Schneider et al. 2007); therefore, it was not unexpected that the fruit and vegetable intake of approximately half of completers in both groups remained < 3 portions per day at the conclusion of the study, with less than 10% of completers in both groups consuming the internationally recommended intake of ≥ 5 portions per day (Naude 2013; WHO 2017). It is possible that the recommendation for increased intake of healthy foods, for example, fruit and vegetables, disseminated in the FBTG sessions may have been difficult to implement by individual participants in our study sample. Love, Maunder and Green (2008) and Solomons, Kruger and Puoane (2017) reported in their qualitative research that some South African population groups consider fruit and vegetables as the more costly items to include regularly in a healthy diet. Temple and Steyn (2011) estimated that an additional R1090.00 per month would be required to cover the costs of healthier food items for a family of five, which is probably not achievable for the majority of participants in our sample who relied on a family income of less than R4166.66 per month. Dietary advice may thus remain difficult to implement if the South African government does not intensify population-scale interventions that reduce cost of healthy foods. At present, it is a significant challenge for health professionals to advise participants to consume healthier, potentially costlier and less palatable foods over possibly less expensive, preferred foods.

The majority of participants in both groups were in ‘contemplation stage’ at baseline, indicating that they may not have been ready to commit to actively changing their diet and increasing their physical activity. The facility-based therapeutic group intervention contributed towards increasing participants’ readiness to change their lifestyle as the proportion of completers in the action stage increased by 43.5% in the FBTG and only by 17.5% in the usual care group over 6 months. Consequently, significantly more FBTG completers were in the action stage at 6 months compared to usual care completers. It should be noted that a quarter of the FBTG completers and half of the usual care completers remained in the contemplation stage, supporting the notion that a greater intervention intensity and/or duration may need to be considered to ensure a more pronounced change in dietary intake, physical activity and weight. It has further been suggested that additional psychological and behavioural interventions, as well as motivational interviewing, may be necessary in resistant participants, especially prior to intensive lifestyle interventions, to enhance movement from a less to a more active SOC (Young 2010). However, the feasibility of implementing these additional strategies in busy PHC settings is not clear.

## Strengths and limitations

In the absence of research on obesity and NCD management in ‘real-world’ settings in PHC facilities in low- and middle-income countries, our findings make an important contribution to insights into feasible options for obesity management. Limitations of our study include the convenience sampling and the non-randomisation of participants as it could contribute to systematic bias. It is possible that patients who volunteered for the FBTG intervention were more motivated, resulting in possible overestimation of the effect of the intervention. However, in PHC settings in South Africa, patients may well be given the choice between usual care and an alternative intervention, with the research design thus simulating this ‘real-world’ situation. A second limitation is the number of participants LTFU, which reduced the sample size and subsequently the power to detect differences between the treatment groups at 6 months. However, the LTFU in both treatment groups (approximately 40%) is within the range of 10% – 80% reported in other weight loss intervention studies (Moroshko et al. 2011). To address this limitation, multiple imputation was conducted to analyse weight change and account for missing data. Finally, the information obtained on dietary intake, physical activity and behavioural measures should be interpreted with caution because it was self-reported.

## Conclusion

The 6-week FBTG intervention implemented at a PHC facility for obese participants with NCDs or risk factors for NCDs may increase the probability of weight loss over 6 months when compared to usual care. Greater improvements in physical activity, dietary intake and readiness for change could contribute to the higher weight loss success in the FBTG completers. Although the weight loss experienced by the FBTG group is statistically significant, it is less than 5% that is sufficient to result in clinically significant improvements in metabolic indicators of NCDs. For this reason, we recommend that future lifestyle interventions for obesity and NCDs in PHC settings should consider making use of the model tested in this research, albeit with a higher intervention dosage and possible down referral of patients from PHC facilities to community-based adherence clubs based on the model of care implemented for HIV-positive individuals on stable antiretroviral therapy in Cape Town (Grimsrud et al. 2016). In addition, a randomised control trial design should be used for future weight loss studies to reduce bias and increase the validity of results.
